# A Prediction Model of Interlayer Bond Strength for 3D-Printed Concrete Considering Printing Interval and Environmental Effects

**DOI:** 10.3390/ma19071377

**Published:** 2026-03-30

**Authors:** Wenbin Xu, Zihao Xu, Tao Liu, Jun Ouyang, Juan Wang, Hailong Wang, Wenqiang Xu

**Affiliations:** 1 College of Water Resources and Civil Engineering, Hunan Agricultural University, Changsha 410128, China; xuwenbin0720@163.com (W.X.);; 2 State Key Laboratory of Hydroscience and Engineering, Tsinghua University, Beijing 100084, China; wangjuan@zzu.edu.cn; 3 Hunan Port Shipping and Water Resources Group Co., Ltd., Changsha 410029, China; 4 College of Civil Engineering and Architecture, Zhejiang University, Hangzhou 310058, China; hlwang@zju.edu.cn

**Keywords:** 3D-printed concrete, interlayer bond strength, printing interval time, environmental factors, moisture variation

## Abstract

Interlayer bond strength is critical for ensuring the safety and durability of 3D-printed concrete (3DPC) structures. However, there remains a lack of real-time prediction methods addressing interlayer performance under the combined effects of interval time and environmental factors during the in situ printing process. To address this issue, this study conducted experiments considering various printing interval times and environmental conditions, incorporating monitoring of dielectric constant and water evaporation, alongside interlayer splitting tensile tests. By integrating the SHAP interpretability algorithm with nonlinear regression analysis, the results indicate that the printing interval time is the dominant factor inducing interlayer strength decay (with a contribution rate of 68.6%), while relative humidity emerges as the primary environmental variable (with a contribution rate of 21.3%). Mechanism analysis reveals that prolonged printing intervals intensify the hydration of the lower deposited layer, leading to reduced interfacial moisture content and loss of plasticity. Furthermore, environmental evaporation significantly regulates this process, with high-humidity environments notably mitigating the moisture loss and strength reduction caused by time delays. Based on the correlation mechanism between moisture and strength, a dimensionless general prediction model for 3DPC interlayer strength was established, incorporating printing interval time and an evaporation index (goodness of fit, R^2^ = 0.96). Consequently, a digital twin quality inversion scheme based on companion specimen monitoring and printing timestamps was proposed. This study quantifies the intrinsic relationships among printing interval time, environmental conditions, and interlayer strength, offering a novel approach for determining the construction window and achieving non-destructive quality prediction for 3DPC in complex environments.

## 1. Introduction

As one of the core technologies for the industrialization and digital transformation of construction, 3D-printed concrete (3DPC) exhibits immense potential in enhancing construction efficiency and realizing complex structures, owing to its moldless forming, high geometric freedom, and automation capabilities [1,2,3]. However, this “layer-by-layer stacking” manufacturing approach introduces unique challenges. The printing process, which eschews traditional formwork and vibration, often leads to the formation of physical interfaces between layers, resulting in air entrapment and geometric discontinuities. The pores and microscopic defects at these interfaces render the interlayer regions mechanically weak points of the component, imparting significant anisotropic characteristics to the structure [4,5,6]. This insufficient interlayer bond strength, commonly referred to as “cold joints,” has become a critical bottleneck constraining the safety and large-scale engineering application of 3DPC structures [7].

The evolution of interlayer bond performance is a complex, time-dependent process involving material rheology, hydration kinetics, and surface physicochemical properties [8,9]. Printing Interval Time (PIT) is a crucial process parameter. Studies indicate that prolonged printing intervals lead to thixotropic reconstruction and early hydration on the surface of the lower concrete layer, altering interface rheology and moisture status. This severely impedes the physical interlocking and chemical bonding between successive layers, resulting in a significant decay in interlayer strength [10,11,12]. Furthermore, 3DPC on-site construction faces complex and variable environmental boundary conditions, such as temperature, humidity, and wind speed [13,14,15]. These environmental factors not only directly affect the hydration rate but also profoundly change the physical state of the interlayer interface by controlling the surface moisture evaporation flux [16,17]. For instance, high temperature and low humidity accompanied by strong winds can drastically accelerate surface moisture loss, leading to premature hardening or drying shrinkage of the interface; conversely, high humidity effectively preserves moisture [18,19]. However, the academic community currently lacks a systematic understanding of how environmental factors quantitatively influence the mechanism of 3DPC interlayer strength, and few studies have integrated variable environmental parameters with printing interval time into a unified analytical framework.

Mechanistically, the quality of interlayer bonding is closely related to the residual moisture content in the contact zone between new and old concrete [20,21]. Existing research primarily explains interlayer weakening mechanisms through microscopic tests (e.g., SEM, MIP, CT), but these are post hoc characterizations, making it difficult to reflect the real-time evolution of interface states during the printing process [22].

Regarding prediction models, current work mainly includes analytical models based on surface moisture and machine learning (ML) based prediction models. For example, Moelich et al. [23] predicted the reduction in interlayer bonding by estimating surface moisture changes. This analytical model achieved an RMSE of 2.5% and an R^2^ of 0.81 in predicting interlayer bond reduction, but its applicability is limited by specific assumptions and a restricted consideration of the comprehensive effects of multiple environmental factors. In recent years, ML models have demonstrated powerful nonlinear mapping capabilities. For instance, Wu et al. [24] developed a stacked ML model that achieved an R^2^ as high as 0.96 and an RMSE of 0.39 MPa for interlayer strength prediction by integrating various printing parameters and material compositions. Abid et al. [25], using an XGBoost model, achieved an R^2^ of 0.998 and an RMSE of 0.066 MPa in predicting interlayer bond strength, demonstrating extremely high prediction accuracy. However, while these ML models exhibit outstanding predictive accuracy, their input features often focus on material composition and printing process parameters (e.g., printing speed, interval time), lacking a systematic decoupled analysis of critical external environmental factors such as ambient temperature, humidity, and wind speed, and their dynamic influence on the interface moisture state. This limitation drives us to seek more accurate theoretical prediction models. Recently, non-destructive techniques such as real-time moisture evaporation monitoring and dielectric constant testing have provided possibilities for quantifying moisture states under environmental effects [26,27], offering new perspectives for revealing the physical essence of interlayer weakening.

Given the current challenges in quantifying the mechanism of environmental effects, identifying multi-factor dominant variables, and real-time non-destructive evaluation on-site, this study aims to establish a comprehensive prediction and evaluation system for 3D-printed concrete interlayer bond strength that considers complex environmental boundary conditions and the evolution of material surface states, in order to overcome the limitations of existing models in generalizing under complex working conditions. To this end, this study constructs a full-chain physical analysis framework for environmental evaporation-interfacial moisture-interlayer strength. Real-time dielectric constant monitoring is employed to precisely quantify the dynamic changes in printing interface moisture. A dimensionless general interlayer strength prediction model, incorporating printing interval and evaporation index, is established. Based on this, a digital twin quality inversion scheme based on companion specimen monitoring is proposed, providing theoretical and technical pathways for determining the construction window and real-time quality warning for 3D-printed concrete under complex climates.

The subsequent structure of this paper is arranged as follows: Section 2 details the full-factorial experimental design covering different printing intervals and combinations of temperature, humidity, and wind speed, as well as the synchronous monitoring methods for dielectric constant and water evaporation. Section 3 innovatively introduces the SHAP (SHapley Additive exPlanations) interpretability algorithm to quantitatively decouple the contributions of printing interval time and environmental parameters to interlayer strength, and deeply analyzes the regulatory mechanism of interfacial moisture evaporation flux on surface plasticity and strength decay. Section 4, based on the aforementioned mechanisms, constructs a dimensionless general interlayer strength prediction model incorporating printing interval time and an evaporation index. Accordingly, a digital twin quality inversion scheme based on companion specimen monitoring is proposed. Finally, Section 5 summarizes the conclusions, clarifying the theoretical basis and technical pathways provided by this study for determining the construction window and achieving non-destructive quality early warning for 3DPC under complex climatic conditions.

## 2. Methodology

To systematically investigate the effects of environmental factors (temperature, humidity, wind speed) and printing interval time on the interlayer bond strength of 3DPC, this study established a comprehensive experimental framework. This section details the methodology adopted, including the preparation of raw materials and the 3D printing process of the specimens. A multi-factor experimental design incorporating various microclimatic conditions and interval times was implemented to simulate different construction scenarios. Furthermore, specific measurement techniques for monitoring early-stage moisture variations and evaluating late-stage mechanical performance are elaborated to ensure the accuracy and reproducibility of the proposed quality prediction model.

### 2.1. Materials and Preparation of 3DPC

#### 2.1.1. Materials

Portland cement (P.O 42.5) was used as the binder, with a specific surface area of 353 m^2^/kg, a density of 3.14 g/cm^3^, an initial setting time of 137 min, and a standard consistency of 26.0%. Its chemical composition is presented in Table 1. The Mix proportion of 3DPC is presented in Table 2. The aggregate consisted of dry river sand with a particle size smaller than 0.1 mm. Mixing water was supplied by the Changsha Water Supply Company. Silica sol, provided by Henghao New Materials Factory, was used, containing a binder content greater than 30% and primarily composed of SiO_2_.nH_2_O. A polycarboxylate superplasticizer (PCE), supplied by Shanghai Chenqi Chemical Co., Ltd., Shanghai, China was employed to improve the fluidity of the fresh concrete. Information regarding the main instruments and equipment used in the experiments is listed in Table 3.

#### 2.1.2. Preparation Process of 3DPC

**Figure 1:**
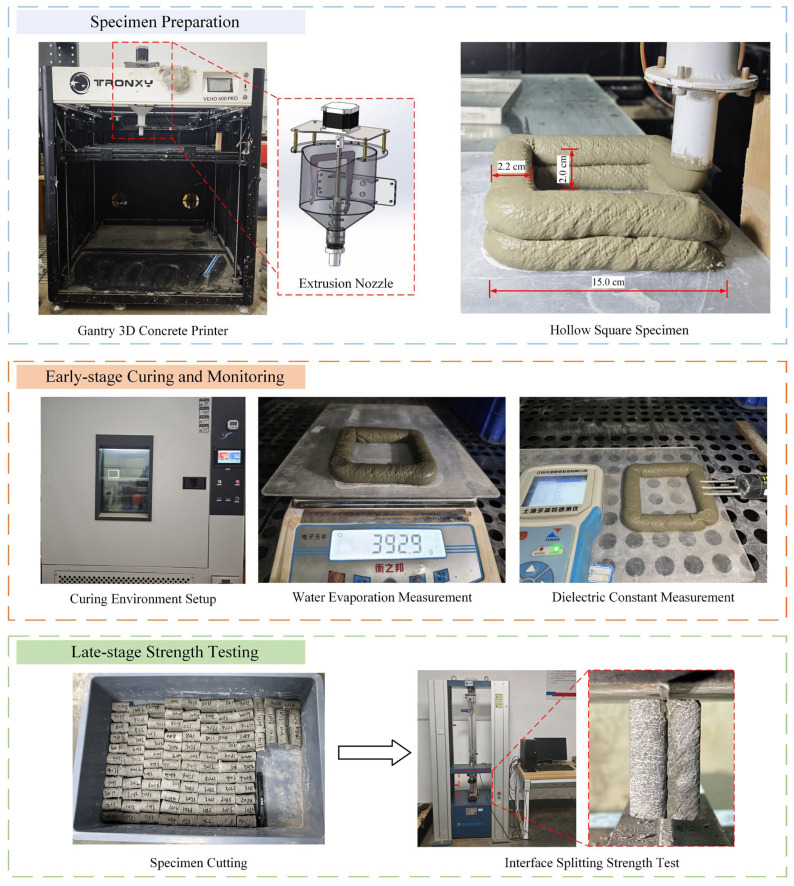
Process of preparation, curing, monitoring, and mechanical performance testing of 3DPC specimens.

In this study, a cement-based material 3D printing system was employed for the preparation of concrete specimens, as shown in Figure 1. The system is primarily a frame structure that utilizes gravity extrusion for 3DPC operations, with a maximum printing volume of 600 mm × 600 mm × 600 mm. The printing speed can be set within the range of 10–150 mm/s, and the operating system is Windows.

Prior to printing, modeling software and slicing software were used for modeling and printing path planning, respectively. After the concrete was thoroughly mixed and homogenized according to the designed mix proportion, it was immediately transferred to the storage hopper of the printer as shown in the figure. The concrete was extruded through the nozzle via screw rotation. The printing speed was set to 30 mm/s, and the layer height was set to 10 mm. As illustrated, the printed specimens used in the experiment were double-layered “square-shaped” structures. The outer side length was 15 cm, and the filament width was 2.2 ± 0.1 cm. The specimens were printed in two layers with a total printing height of 2.0 cm.

### 2.2. Multi Factors Experimental Scheme

#### 2.2.1. Environmental Factors

As shown in Table 4, eight environmental conditions were established in this study, corresponding to different combinations of temperature, humidity, and wind speed. Specifically, temperatures included a high temperature of 30 °C and a low temperature of 10 °C; humidity levels considered high humidity (RH = 85%) and low humidity (RH = 55%); and wind speeds considered a windless condition and a wind speed of 2 m/s. A programmable constant temperature and humidity chamber was used to regulate the environmental parameters, with a temperature adjustment range of 20–95 °C (precision: 1.0 °C) and a humidity adjustment range of 45–95% (precision: 1.0%). Wind speed was controlled by a fan with an adjustment range of 0–6 m/s; precise control of the wind speed relied on an anemometer, which had a measurement range of 0.05 to 5.00 m/s and a precision of 0.01 m/s.

#### 2.2.2. Printing Interval Time

The printing interval time significantly impacts the interlayer performance of 3D-printed concrete. In this study, different printing interval times were set to investigate their influence, along with environmental conditions, on interlayer performance. In the experiments, the printing interval time was varied in 10 min increments, covering a total duration of 2 h, resulting in 13 distinct time points, as detailed in Table 5. Specifically, “0 min interval” refers to the shortest actual interval from the completion of the first layer to the start of the second layer (approximately 20 s), used to simulate the most ideal bonding condition. The extended interval, covering up to 120 min, aims to systematically reveal the decay pattern of interlayer bond performance, providing data support for potential extreme situations such as printing interruptions. This range ensures the universality of the research findings across different construction speeds and scenarios. The specific settings for the interval times are presented in Table 5.

Considering that the printer could not be placed inside the constant temperature and humidity chamber, this study investigated the environmental impact on interlayer performance by transferring the specimens after printing. The operation procedure was as follows: immediately after the first layer of concrete was printed, the sample was placed into the chamber set to the specified environmental conditions. Once the designated printing interval time was reached, the sample on the printing tray was transferred back to the 3D printing platform for the deposition of the second layer. After the printing process was fully completed, the specimen was transferred back to the chamber. To minimize disturbance to the specimens during transfer, the environmental chamber was positioned immediately adjacent to the 3D printer. The transfer process of the specimens was extremely brief, typically controlled within a few seconds.

### 2.3. Measurement Methods

#### 2.3.1. Dielectric Constant Measurement

To characterize the surface moisture distribution of the printed filaments, this study adopted the method of measuring the dielectric constant of concrete. In the experiment, an independent single-layer specimen was printed separately under identical environmental conditions and immediately transferred to the constant temperature and humidity curing chamber upon completion. The measurement employed a three-parameter portable measuring instrument equipped with a high-precision digital TDR sensor, as shown in Figure 1. The probe length was 150 mm, with a dielectric constant measurement range of 1–80, an accuracy of ±1%, and a response time of 0.25 s. To minimize measurement contingency, the dielectric constant was measured three consecutive times at each set printing interval point, and the average value was taken as the representative value for that interval.

#### 2.3.2. Water Evaporation Measurement

To quantify the cumulative water evaporation of the specimens over time, this study monitored the mass loss of the printed blocks. During the experiment, an independent single-layer specimen was printed separately under identical environmental conditions and immediately transferred to the constant temperature and humidity curing chamber upon completion. An electronic balance with a precision of 0.1 g was used to measure the mass of the block, recording the initial mass as $m_{0}$. During the evaporation measurement process, the mass of the block was recorded as $m_{t}$ every 10 min. The formula for calculating the cumulative evaporated water amount $W_{z}(t)$ at different printing interval points is:

(1) $$W_{z}\left(t\right)=m_{0}-m_{t}$$

Based on the initial mass of the specimen and the mix proportion, the initial water content $W_{0}$ of the specimen could be calculated. The ratio of the evaporated water amount to the initial water content is defined as the evaporation moisture index $\alpha_{E}$, used to characterize the degree of moisture evaporation from the specimen:

(2) $$\alpha_{E}=\frac{W_{z}}{W_{0}}$$

#### 2.3.3. Interlayer Mechanical Performance Measurement

To evaluate the interlayer bond performance of 3D-printed concrete, direct tensile or shear tests are commonly employed. However, considering that tensile tests are susceptible to eccentric effects during specimen preparation, and shear tests often lead to mixed failure due to strength disparities, making it difficult to obtain pure interfacial strength [7,28]. This study opted for the splitting tensile test. Firstly, this method is recommended by the Chinese 3D-Printed Concrete Standard (T/CECS 786-2020) and has been adopted in several studies [25]. Secondly, compared to direct tensile testing, splitting tensile testing offers significant advantages in terms of sample preparation and testing efficiency, facilitating large-scale parameter studies. Although splitting tensile testing introduces a complex stress field, the principal tensile stress induced in the central region of the interlayer is perpendicular to the interface, effectively promoting tensile failure at the interface. Based on normative guidance, operational convenience, and effective characterization of interfacial tensile properties, this study believes that the splitting tensile test can reliably reflect interlayer bond strength.

All specimens to be tested were prepared using the cement-based material 3D printing system. After curing for 14 days under standard curing conditions, the specimens were processed into specific dimensions via precision cutting and polishing: length of 50 mm, width of 22 ± 1 mm, and height of 20 mm, as shown in Figure 1. The experimental design covered 8 environmental conditions and 13 printing interval time points, totaling 104 sets of experimental conditions. Five specimens were selected for testing in each condition to ensure data reliability. The test employed a microcomputer-controlled electronic universal testing machine (capacity 10 kN, precision 0.1 N), with displacement control loading at a rate of 0.01 kN/s. The calculation formula for the interlayer splitting tensile strength F is as follows:

(3) $$F=\frac{2P}{\pi A}$$

where P is the failure load, and *A* is the splitting area.

It should be noted that for the companion specimens used for continuous monitoring of dielectric constant and evaporation, once printed and transferred to the environmental chamber, they remained undisturbed for continuous monitoring. However, for the specimens designated for interlayer splitting tensile strength testing, it was necessary to remove them from the environmental chamber after the set interval time for re-printing, and then return them to the environmental chamber. Thus, these specimens underwent two transfer processes.

## 3. Results

### 3.1. Dielectric Constant Results

**Figure 2:**
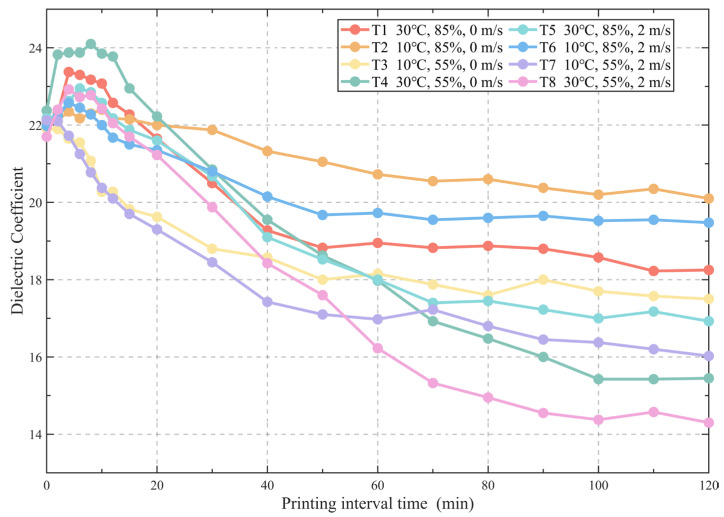
Evolution law of dielectric constant of 3DPC with printing interval time under different environmental conditions.

The variation in the dielectric constant of 3DPC with printing interval time under different environmental conditions is shown in Figure 2. As can be seen from the figure, in the initial stage (within 20 min), the dielectric constant changes drastically, exhibiting an overall trend of first increasing and then decreasing. This indicates significant fluctuations in the surface moisture content of the concrete during the early printing phase: moisture increases rapidly within the first 5 min, exhibiting a surface bleeding phenomenon; subsequently, it remains relatively stable for approximately 2 to 10 min; thereafter, the surface moisture begins to decline rapidly. This bleeding process is consistent with the experimental results of Moelich et al., who defined it as the second stage of surface moisture evolution in 3DPC in their related research [23].

In the stage from 20 to 70 min, the dielectric constants under different working conditions all gradually decrease, but the rate of decline is significantly influenced by environmental conditions. Specifically, under the conditions of 55% humidity, 30 °C temperature, and 2 m/s wind speed, the dielectric constant drops the fastest, decreasing from 21.2 to 15.3. Conversely, under the conditions of 85% humidity, 10 °C temperature, and 0 m/s wind speed, the decline rate is the slowest, with the dielectric constant dropping only from 22.0 to 20.5, representing a minor reduction. This demonstrates that as time progresses, the surface moisture content of 3DPC gradually diminishes, and more adverse environmental conditions accelerate this process. After 70 min, under the continuous influence of hydration reactions and environmental factors, the dielectric constant continues to decrease, but the trend of change tends to plateau.

### 3.2. Water Evaporation Results

**Figure 3:**
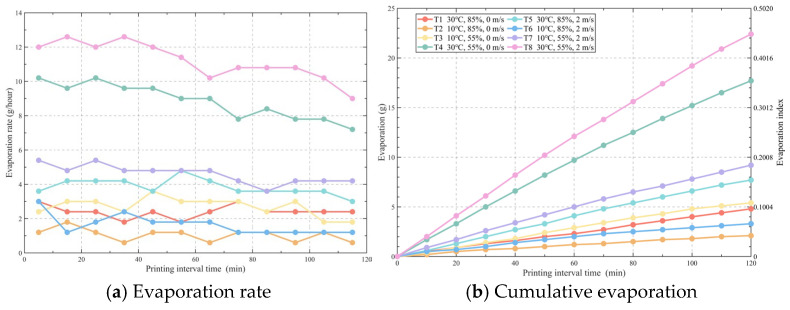
Evolution law of evaporation behavior of 3DPC with printing interval time under different environmental conditions: (**a**) Evaporation rate; (**b**) Cumulative evaporation.

The variations in evaporation rate and cumulative evaporation of 3DPC with printing interval time under different environmental conditions are shown in Figure 3. Figure 3a indicates that the evaporation rate of the specimens is closely related to the printing environment. Under the harsh environmental conditions of T8, the evaporation rate reaches a maximum of 12 g/h, whereas under the more favorable conditions of T2, the evaporation rate is only 1 g/h, representing a nearly tenfold difference. Furthermore, within the first two hours of testing, the evaporation rates of most specimens remain basically stable; only under the high-evaporation conditions of T8 and T4 does the evaporation rate show a slight decrease after 70 min.

Regarding cumulative evaporation, there is a clear correlation between the amount of evaporation and various environmental conditions. When environmental humidity decreases, temperature rises, or wind speed increases, the cumulative water evaporation increases correspondingly. Specifically, under the conditions of 55% humidity, 30 °C temperature, and 2 m/s wind speed (Condition T8), the evaporation is the highest, with a cumulative evaporation of 22.4 g at 120 min. Conversely, under the conditions of 85% humidity, 10 °C temperature, and 0 m/s wind speed (Condition T2), the evaporation is the lowest, with a cumulative evaporation of only 2.1 g at 120 min, still showing an approximately tenfold difference between the two.

According to the evaporation index on the right side of Figure 3b, under conditions T8 and T4, the evaporation indices are 0.45 and 0.35, respectively. This indicates that at 120 min after printing completion, the initial water content of the concrete has been lost by 45% and 35%, respectively. Under the remaining conditions, the moisture evaporation loss over the same period is all below 20%.

### 3.3. Splitting Tensile Strength Results

**Figure 4:**
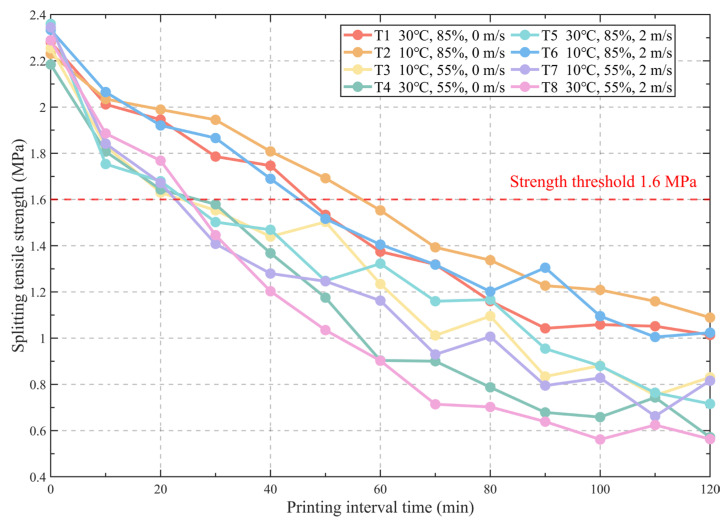
Evolution law of late-age splitting tensile strength of 3DPC with printing interval time under different environmental conditions.

The variations in the late-age splitting tensile strength of 3DPC with printing interval time under different environmental conditions are shown in Figure 4. It can be seen from the figure that the splitting tensile strength under different environmental conditions exhibits an overall monotonic downward trend with printing interval time, indicating that the prolongation of the printing interval continuously weakens the interlayer interface bond performance. At the initial moment (0 min), the splitting tensile strength of each working condition is basically at the same level of approximately 2.2–2.4 MPa. When the printing interval increases to about 60 min, the strength of most test groups has decayed to the range of approximately 1.0–1.6 MPa, and the differences between different environmental conditions are significantly amplified. When the interval is further extended to 120 min, the strength drops to about 0.6–1.1 MPa. The 1.6 MPa strength threshold set in the figure indicates that most working conditions successively fall below this level within the 20–60 min range, reflecting that the acceptable printing interval time is relatively limited.

Regarding environmental factors, under relatively mild conditions of high humidity and no wind (e.g., T1: 30 °C, 85%, 0 m/s), the strength decay is relatively slow; the curve falls below the reference threshold of 1.6 MPa only at around 40–50 min, and remains at about 1.1 MPa at 120 min. In contrast, working conditions with low humidity (e.g., T3, T4, T7, T8) exhibit faster strength degradation, falling below the 1.6 MPa threshold within about 20–30 min, and the strength can drop to a minimum of about 0.6–0.8 MPa at 120 min. The strength of the T5 condition (high humidity but high temperature and wind) under long printing intervals is also significantly lower than its windless or low-temperature counterparts, indicating that introducing airflow under high-temperature conditions significantly aggravates convective drying and moisture loss in the interfacial region, thereby further weakening the interlayer bond performance. Combining with water evaporation results, it is evident that reducing relative humidity and increasing wind speed both accelerate interlayer interface moisture loss and early surface hardening, weakening the mechanical interlocking and interfacial hydration degree between new and old layers. Consequently, under the constraint of the given strength threshold (1.6 MPa), the acceptable printing interval time is significantly shortened, demonstrating that both printing interval time and environmental conditions significantly affect the interlayer splitting tensile strength of 3DPC.

### 3.4. Multi-Factor Analysis

#### 3.4.1. Qualitative Analysis

To deeply understand the independent contribution and potential nonlinear effects of each input variable (printing interval time, ambient temperature, ambient humidity, ambient wind speed) on interlayer splitting tensile strength, this study adopted the SHAP (SHapley Additive exPlanations) method for qualitative analysis, referencing the research by M Kovačević et al. [29]. SHAP is a local interpretable model-agnostic (LIME) method based on cooperative game theory, capable of quantifying each feature’s contribution to model predictions and revealing its influence direction on the prediction results, thereby significantly enhancing the transparency of variable impact analysis.

It is important to note that the calculation of SHAP values is based on a trained Random Forest Regressor model. The Random Forest model was chosen for its ability to effectively capture nonlinear relationships and potential interactions between features, providing a robust explanatory basis for SHAP. Prior to conducting SHAP analysis, we first divided the dataset into training and testing sets (80% for training, 20% for testing). Subsequently, the Random Forest model was trained using the training data (X_train, i.e., all input features; y_train, i.e., the target value of interlayer splitting tensile strength). To balance model performance with interpretability and avoid overfitting, the key hyperparameters of the Random Forest model were optimized: the number of decision trees was set to 300, the maximum depth of a single decision tree was limited to 5 layers to prevent overfitting, and each node split required at least 5 samples to ensure the reliability of the split.

In the subsequent SHAP analysis, no explicit variable selection steps were performed. All original input features (printing interval time, ambient temperature, ambient humidity, ambient wind speed) were included in the Random Forest model for training and then interpreted by SHAP to comprehensively evaluate their contributions to interlayer splitting tensile strength. The performance metrics of the Random Forest model on the training and testing sets are as follows: training set R^2^ was approximately 0.98, RMSE was approximately 0.03, and MAE was approximately 0.02; testing set R^2^ was approximately 0.92, RMSE was approximately 0.06, and MAE was approximately 0.05. These results indicate that the Random Forest model performs well in capturing the intrinsic relationships within the data and exhibits good generalization ability on unseen data. The calculated SHAP importance contribution rates and their percentages for each factor are shown in Table 6. These results reflect the average absolute contribution of each feature to the interlayer strength prediction within the trained Random Forest model.

As indicated in Table 6, interval time is the decisive factor affecting interlayer bond strength, with a SHAP contribution rate of 0.4254, corresponding to 68.6%. Humidity is the second most important factor, with a contribution rate of 0.1318, accounting for approximately 21.3%. The independent contributions of temperature and wind speed are relatively small, with contribution rates of 0.0415 (6.7%) and 0.0211 (3.6%), respectively. Based on the SHAP results, it can be analyzed that the interlayer interval time dominates the evolution of interfacial moisture and the internal microstructure of concrete through cumulative effects, acting as the primary factor for strength decay. Environmental parameters regulate the rate and magnitude of this cumulative process by altering evaporation flux and moisture content.

The printing interval time has a significant impact on the late-age interfacial splitting tensile strength. This may be because 3DPC, as an early-strength system, undergoes extremely intense hydration reactions and moisture migration in the initial stage. The moisture content, structural build-up, and remoldability at the interface evolve rapidly with time. As the interval time extends, the interface gradually transforms from a high-moisture, reshapeable state to a low-moisture, hardened state, thereby leading to a significant reduction in splitting tensile strength.

The contribution of humidity is higher than that of temperature and wind speed mainly because it directly controls the vapor pressure deficit between the air and pores, thereby exponentially altering the evaporation flux and internal relative humidity. Experimental results show that the cumulative evaporation under low humidity conditions is about 10 times that under high humidity conditions at 120 min, having the most significant effect on material moisture content and interfacial wettability. High humidity environments effectively inhibit moisture escape, delay the formation of cold joints, and retain a certain window for wetting and re-bonding.

In addition, increasing temperature enhances evaporation potential on one hand, and accelerates early hydration and structural build-up on the other, thereby shortening the interfacial printable time to a certain extent. However, within the range of 10–30 °C in this study, its impact magnitude on interfacial evolution is limited, so its independent contribution is lower than that of time and humidity. Wind speed mainly promotes evaporation by enhancing convective mass transfer. However, under the wind speed and vapor pressure deficit levels in this experiment, its influence on overall evaporation and interfacial state is relatively weak, thus exhibiting the lowest contribution rate in the SHAP analysis.

In summary, the SHAP analysis quantitatively verified the experimental phenomena: interval time is the primary factor controlling the evolution of interlayer moisture and microstructure and is the main control variable for interlayer strength decay. Humidity has a significant effect on interlayer splitting tensile strength by regulating the evaporation driving force and is the leading factor among environmental factors. Within the working conditions of this study, the effects of temperature and wind speed on evaporation and interfacial state are relatively secondary, but under adverse combinations (e.g., high temperature, windy), they will still further aggravate the deterioration of interlayer performance.

#### 3.4.2. Quantitative Characterization

**Figure 5:**
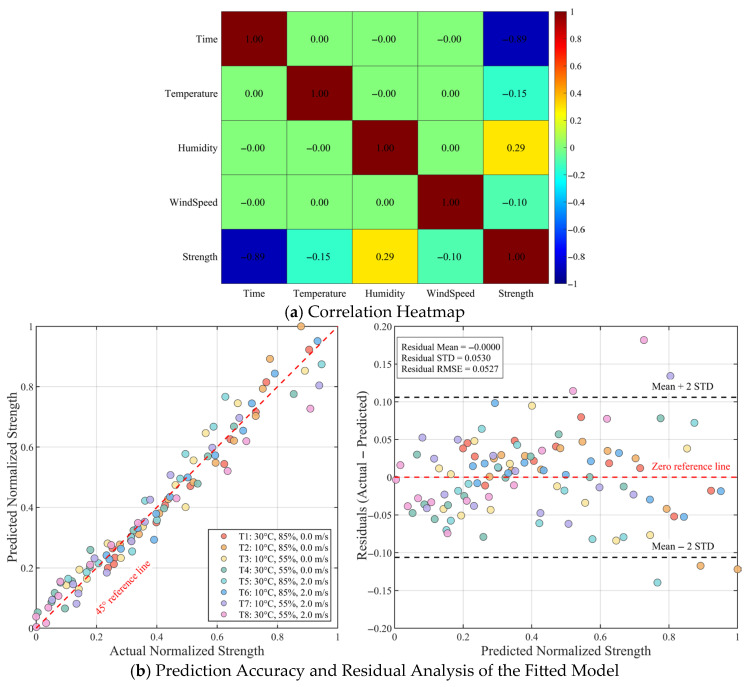
Correlation and model accuracy analysis for printing interval time, humidity, temperature, wind speed, and split-ting tensile strength: (**a**) Correlation Heatmap; (**b**) Prediction Accuracy and Residual Analysis of the Fitted Model.

To gain a deeper understanding of the linear relationships among the influencing factors (printing interval time, ambient temperature, ambient humidity, ambient wind speed) on interlayer splitting tensile strength, and to provide intuitive basis for the subsequent construction of multivariate regression models, this study first conducted a Pearson correlation analysis between all input variables and the output variable (interlayer splitting tensile strength). As shown in Figure 5a, the Pearson correlation coefficient matrix between variables clearly reveals the strength and direction of their linear associations. The analysis results indicate a significant strong negative correlation between printing interval time and interlayer splitting tensile strength (r = −0.89), which is highly consistent with the earlier mechanistic analysis that prolonged printing intervals lead to a noticeable decrease in strength. Ambient humidity exhibits a weak positive correlation with interlayer splitting tensile strength (r = 0.29), suggesting that an increase in humidity within a certain range may help mitigate strength loss. Conversely, ambient temperature and ambient wind speed show relatively weaker correlations with interlayer splitting tensile strength, with r = −0.15 and r = −0.10, respectively, both being negative, indicating a slight negative impact of increased temperature or wind speed on strength. Consistent with the previous SHAP analysis results, these correlation analyses provide a foundation for further constructing multivariate prediction models and help explain the independent directional effects of each variable on interlayer strength.

Based on the aforementioned mechanistic understanding, this study shifts to a direct fitting tool for engineering applications, employing a multivariate regression method. A prediction model for interlayer splitting tensile strength, varying with printing interval time and environmental parameters, is established by fitting the entire experimental dataset using a combination of a quadratic time term and first-order environmental parameter terms. In data processing, actual measured values were used for interlayer splitting tensile strength, humidity, temperature, and wind speed. Considering that interval time is the dominant factor and its influence on strength exhibits a clear nonlinear decay, a quadratic polynomial form was adopted for the time term, while linear terms were used for temperature, humidity, and wind speed. The resulting strength prediction model can be expressed as a clear, easily implementable formula for engineering, facilitating rapid application in determining design windows, construction scheduling, and process optimization. The fitted strength prediction model can be expressed as:

(4) $$\begin{array}{l} F\left(t,x,y,z\right)=0.2948t^{2}-1.2766t-0.0073x+0.93y-0.0461z+1.7431\\ R^{2}=0.9565 \end{array}$$

where F is the interlayer splitting tensile strength, MPa; t is the printing interval time, h; x is the environmental temperature, °C; y is the environmental humidity, % (range [0, 1]); and z is the environmental wind speed, m/s.

The coefficient of determination ($R^{2}$) of the model is 0.9565, and the overall error is within an acceptable range, indicating that the model can well characterize the variation law of interlayer strength under the action of multiple factors. Specifically, the fitted coefficient for the quadratic time term reflects a significant nonlinear decay of strength with increasing printing interval time, which aligns strongly with the pronounced negative correlation shown in Figure 5a and the experimental phenomena it reveals. Concurrently, the signs of the coefficients for environmental humidity, temperature, and wind speed terms in the model are consistent with the linear correlation directions presented in Figure 5a. This further quantitatively reflects the independent contribution of these environmental parameters to the interlayer bonding performance: increasing humidity helps improve strength, while increasing temperature or wind speed weakens bonding performance. This demonstrates that the multivariate regression model can quantitatively reflect the decay law of interfacial strength under the multi-factor coupling effects of printing interval time and environmental parameters.

It needs to be clarified that the R^2^ values mentioned here and subsequently refer to the goodness of fit for the entire dataset, not the generalization assessment metric of the training set. The figures will clearly present the numerical values of the fitting parameters, as well as the distribution characteristics of the fitting residuals and their relationship with the predicted strength. The proposed fitting model aims to provide a simple, easy-to-use, and interpretable tool to assist engineers in rapidly quantifying the combined effects of printing intervals and environmental factors under field conditions.

Figure 5b presents the prediction accuracy and residual distribution of the fitted model based on Equation (4) under various working conditions. As shown in Figure 5b, the predicted normalized strength and the measured normalized strength are generally distributed uniformly along the y=x reference line. Most data points are highly concentrated near the diagonal, indicating that the model maintains high prediction accuracy under different combinations of printing intervals and environmental conditions. Figure 5b shows the variation in residuals (measured value minus predicted value) with predicted normalized strength: the residuals are distributed approximately symmetrically around 0, with a mean close to 0 and a standard deviation of about 0.053. The vast majority of residuals fall within the ±2σ standard deviation range, and no trend of systematic deviation with increasing predicted strength is observed. This indicates that the multivariate fitting model does not have significant systematic errors or range-correlated biases and can be reliably used to quantitatively evaluate the interlayer splitting tensile strength under given printing interval time and environmental parameter conditions.

## 4. Discussion

### 4.1. Time–Evaporation–Strength Model

The previous results based on SHAP analysis and multivariate regression indicate that the printing interval time, together with environmental parameters such as temperature, humidity, and wind speed, controls the evolution of interlayer splitting tensile strength. Among these, time is the dominant factor, and humidity is the most important environmental factor. However, such data-driven models primarily reflect empirical correlations. The contribution of each variable to strength is expressed as a statistical “weight,” which makes it difficult to directly reveal the unified underlying physical processes, especially the intrinsic relationship between moisture changes and interfacial strength development. Therefore, it is necessary to further construct a strength model in the Discussion Section based on hydration and water content. This model aims to integrate evaporation loss, effective hydration water, and interfacial bonding capacity under different environmental conditions into a unified framework, thereby interpreting and extending the experimental phenomena and regression results from a mechanistic level.

Since 3DPC is a typical layered casting material, assuming a reasonable mix design, all water in the initial mix proportion can be considered effective water for the hydration reaction process, and the development of interlayer strength mainly stems from the cementation produced by the hydration reaction.

**Figure 6:**
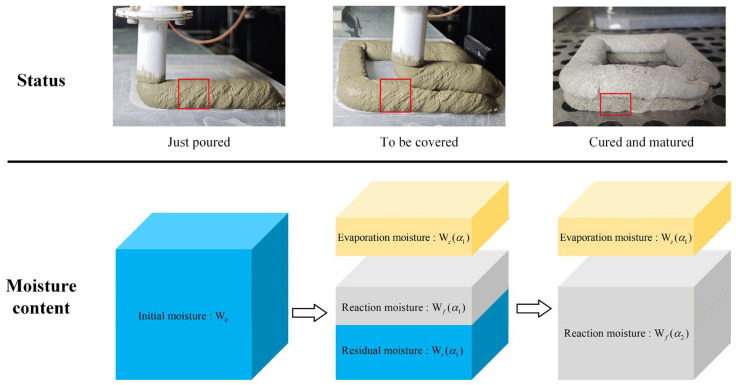
Schematic diagram of moisture change in 3DPC.

As shown in Figure 6, based on the state of 3DPC at different stages, its hydration process can be divided into the following three phases:

(1) The first phase is the moment immediately after printing is completed, at which time the initial water content of the concrete is $W_{0}$.

(2) The second phase is the moment just before being covered by the upper layer of concrete. At this point, the water in the concrete can be divided into three parts: evaporated water $W_{z}(\alpha_{1})$, chemically bound water consumed by reaction $W_{f}(\alpha_{1})$, and remaining water $W_{r}(\alpha_{1})$. At this stage, the degree of hydration $\alpha_{1}$ of the 3DPC matrix can be expressed as:

(5) $$\alpha_{1}=\frac{W_{f}(\alpha_{1})}{W_{0}}=\frac{W_{0}-W_{r}(\alpha_{1})-W_{z}(\alpha_{1})}{W_{0}}$$

(3) The third phase is the moment when the hydration reaction is basically completed. At this time, the water has only transformed into two parts: evaporated water $W_{z}(\alpha_{2})$ and chemically bound water consumed by reaction $W_{f}(\alpha_{2})$. It should be noted that after the second layer of concrete is printed, the surface of the lower layer is covered, and evaporation stops, meaning $W_{z}(\alpha_{2})=W_{z}(\alpha_{1})$. At this point, the degree of hydration $\alpha_{2}$ can be expressed as:

(6) $$\alpha_{2}=\frac{W_{f}(\alpha_{2})}{W_{0}}=\frac{W_{0}-W_{z}(\alpha_{1})}{W_{0}}$$

Regarding the strength of the reaction zone, existing studies [30,31,32] have shown that the relationship between strength *F* and the degree of hydration *α* is as follows:

(7) $$\frac{F}{F_{0}}={\left[\frac{\left(\alpha -\alpha_{0}\right)}{\left(1-\alpha_{0}\right)}\right]}^{b}$$

where $F_{0}$ is the maximum strength achievable by the concrete under the original mix proportion when hydration is complete, which corresponds to the matrix strength in 3DPC; $\alpha_{0}$ is the critical degree of hydration when the concrete just begins to develop strength, which is a constant value for a determined material mix; and b is an exponential coefficient.

Regarding the interlayer bond strength, before the second layer of concrete is printed, the surface of the first layer has not yet formed effective strength, meaning the initial interlayer strength is zero. However, after the second layer is printed and enters the curing stage, it can be assumed that the growth rate of the interlayer strength is consistent with that of the bulk strength of the lower layer concrete.

Therefore, the total increment of interlayer bond strength ΔF can be defined as the strength development from the moment of the second layer casting (corresponding to hydration degree $\alpha_{1}$) to the moment of hydration completion (corresponding to hydration degree $\alpha_{2}$), that is:

(8) $$\Delta F=\frac{F_{0}}{{\left(1-\alpha_{0}\right)}^{b}}\left[{\left(\alpha_{2}-\alpha_{0}\right)}^{b}-{\left(\alpha_{1}-\alpha_{0}\right)}^{b}\right]$$

Substituting Equations (5) and (6) into Equation (7), the expression for interlayer strength ΔF can be derived.

Equation (8) establishes a quantitative relationship between interlayer strength, evaporated water ($W_{z}$), and remaining water ($W_{r}$). Among them, the cumulative evaporated water $W_{z}$ has been determined through experiments, while the remaining water $W_{r}$, as an internal state variable not directly observed, needs to be based on the law of conservation of water mass. The value of $W_{r}$ depends on the competition between physical evaporative water loss and chemical hydration water consumption. However, for a given mix proportion, the chemically bound water $W_{f}$ is mainly determined by hydration kinetics and can be regarded as a function of time, while $W_{z}$ is driven by environmental conditions. To simplify the model, assuming that within the printing interval time, the surface physical evaporation process and the internal chemical hydration reaction are kinetically approximately decoupled (i.e., proceed independently without mutual interference), the remaining water can be further expressed as the residual value of the initial water after deducting evaporation and hydration consumption, expressed in terms of time and evaporated water:

(9) $$W_{r}=f_{1}(t)+f_{2}(W_{z})$$

Analysis of the experimental results indicates that among the factors affecting interlayer strength, time (T) is the primary factor, and environmental evaporation ($W_{z}$) is the secondary factor. Based on the physicochemical characteristics of cement hydration reactions, the reaction rate is highest in the initial stages and then gradually slows down, exhibiting a trend of nonlinear decay. Therefore, employing a linear term to describe the effect of printing interval time on interlayer strength is inappropriate. Compared to a linear term, a quadratic function can better capture this nonlinear characteristic, which involves an initial rapid decrease followed by a gradual leveling off. While cubic or other higher-order terms might mathematically offer finer fitting, introducing too many parameters would not only significantly increase model complexity, leading to a higher risk of overfitting, but also provide limited improvement in accuracy given our current data scale. Consequently, a quadratic function offers the optimal balance between physical reasonableness and model simplicity in describing the temporal effect. Thus, the time function can be set as a quadratic function, and the evaporation function as a linear function. By setting b=1 in the simplified model [33,34], the following can be obtained:

(10) $$\Delta F=\frac{F_{0}}{\left(1-\alpha_{0}\right)}\cdot \frac{W_{r}(a_{1})}{W_{0}}=m_{1}\cdot T^{2}+m_{2}\cdot T+m_{3}\cdot W_{z}+m_{4}$$

where $m_{1},m_{2},m_{3},m_{4}$ are coefficients to be fitted, which can be determined through experiments. Based on this, this paper proposes a prediction model for the interlayer strength of 3DPC with printing interval time and evaporation amount as independent variables.

### 4.2. Model Validation

**Figure 7:**
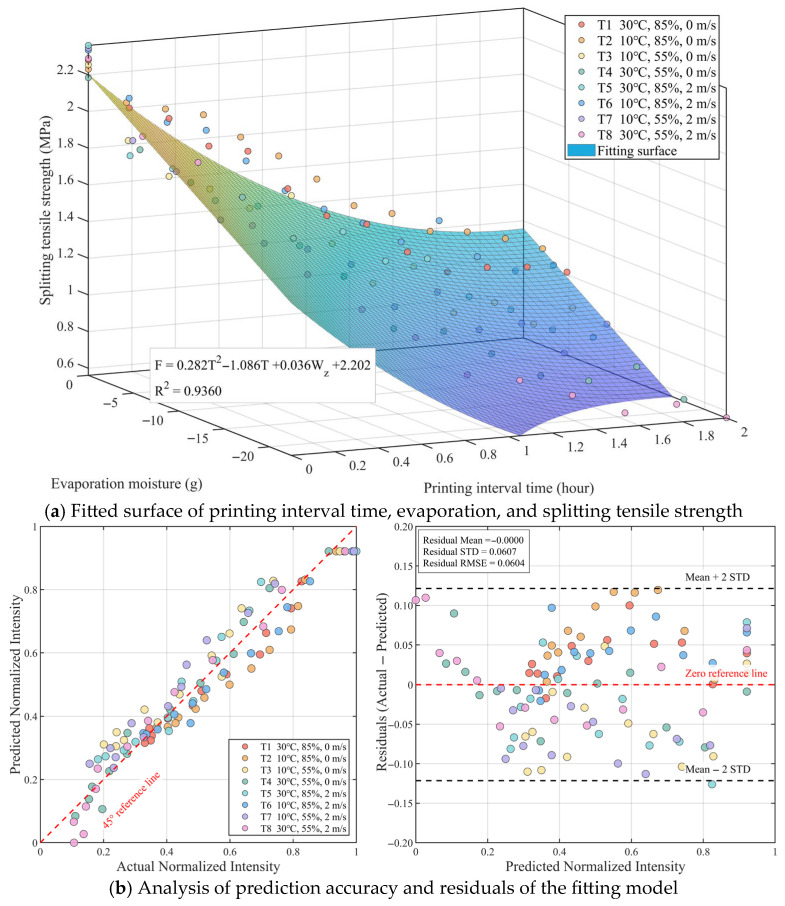
Regression fitting and model validation of concrete splitting tensile strength based on quadratic time term and lin-ear evaporation term: (**a**) Fitted surface of printing interval time, evaporation, and splitting tensile strength; (**b**) Analysis of prediction accuracy and residuals of the fitting model.

Based on the established theoretical model for interlayer strength, the experimentally measured interlayer splitting tensile strength, printing interval time, and cumulative evaporation under corresponding conditions (input as negative values to represent moisture loss) were substituted into the equation. Multivariate non-linear regression analysis was performed using the least squares method. The fitted response surface and the specific regression equation are shown in Figure 7a. The regression results show that the interlayer splitting tensile strength (F) satisfies the following relationship with the printing interval time (T) and evaporated water ($W_{z}$):

(11) $$\begin{array}{l} F=0.282T^{2}-1.086T+0.036W_{z}+2.202\\ R^{2}=0.9360 \end{array}$$

The coefficient of determination ($R^{2}$) of the model reaches 0.9360, indicating that this semi-empirical and semi-mechanistic model possesses a high goodness of fit. Analyzing the physical significance of the regression coefficients, the linear coefficient of the time term is −1.086 while the quadratic coefficient is 0.282. This parabolic form opening upwards accurately characterizes the non-linear feature where interlayer strength monotonically decays with time but at a gradually decreasing rate. This is consistent with the experimental phenomenon observed in Figure 7, where strength drops rapidly with time and then tends to plateau. For the evaporation term, the coefficient is 0.036. Since the evaporation amount $W_{z}$ in the input data is a negative value, this positive coefficient implies that as the absolute value of water evaporation increases, the calculated strength decreases. This quantitatively verifies from a mechanistic perspective the observed law that high-evaporation working conditions (such as T8) lead to more severe strength loss, confirming the physical hypothesis that environmental factors weaken interlayer bonding capacity by aggravating water evaporation.

Figure 7b further displays the error statistical characteristics of the model. The high agreement between the predicted normalized strength and the measured values near the 45° line demonstrates the good generalization ability of the model. The statistical results show that the mean residual approaches 0, and the root mean square error (RMSE) and standard deviation (STD) are 0.0604 and 0.0607, respectively. Furthermore, the vast majority of residuals fall within the ±2σ standard deviation interval and are randomly distributed, with no obvious heteroscedasticity or systematic bias observed. This result confirms that introducing evaporation amount to represent environmental influence into the physical model not only makes it more interpretable in terms of mechanism but also provides sufficient statistical accuracy to reliably predict interlayer bond performance under complex working conditions.

The aforementioned evaporation-based model has clarified the evolution mechanism of interlayer strength from the perspective of water mass conservation. To further verify this law from the perspective of material physical state and explore portable detection methods suitable for on-site applications, this paper attempts to introduce the dielectric constant as another key state variable to construct a prediction model. The dielectric constant is highly sensitive to the internal free water content of the material and can non-destructively reflect the real-time wetting state of the concrete surface, serving as an effective supplement and cross-verification for evaporation data.

**Figure 8:**
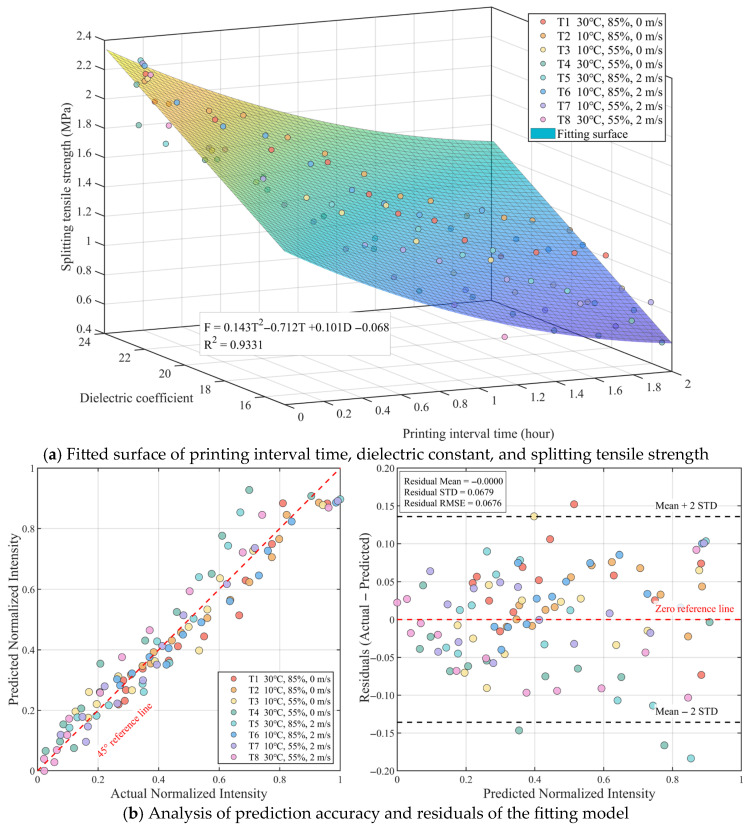
Regression fitting and model validation of concrete splitting tensile strength based on quadratic time term and lin-ear dielectric constant term: (**a**) Fitted surface of printing interval time, dielectric constant, and splitting tensile strength; (**b**) Analysis of prediction accuracy and residuals of the fitting model.

Substituting the experimentally measured dielectric constant D into the model instead of the evaporation amount for non-linear regression, the results are shown in Figure 8a. The interlayer splitting tensile strength (F) satisfies the following relationship with the printing interval time (T) and dielectric constant (D):

(12) $$\begin{array}{l} F=0.143T^{2}-0.712T+0.101D-0.068\\ R^{2}=0.9331 \end{array}$$

Its coefficient of determination ($R^{2}$) is as high as 0.9331, which is comparable to the precision of the evaporation model. Specifically, the coefficient of the dielectric constant term is 0.101, indicating that at the same time interval, a higher dielectric constant corresponds to more sufficient surface free water, which is conducive to interlayer hydration cementation. The error analysis in Figure 8b further shows that the root mean square error (RMSE) of this model is only 0.0676, and the residuals are uniformly distributed without systematic bias. This indicates that the dielectric constant and evaporation amount have good consistency in characterizing the interlayer moisture state. The model based on dielectric properties not only corroborates the correctness of the hydration-evaporation theory but also provides a highly potential non-destructive testing alternative for evaluating interlayer strength in engineering scenarios where direct weighing is difficult.

### 4.3. Model Optimization

Although the aforementioned model employing a linear term for evaporation has successfully revealed the evolution law of interlayer strength, the non-linear characteristics of the experimental data become increasingly significant when dealing with high-evaporation working conditions (such as T8). Consequently, the linear assumption might introduce specific prediction deviations under extreme conditions. To further enhance the prediction accuracy and applicable scope of the model, this paper attempts to incorporate a quadratic term for evaporation into the original physical model, constructing an optimized model with a higher degree of nonlinearity.

**Figure 9:**
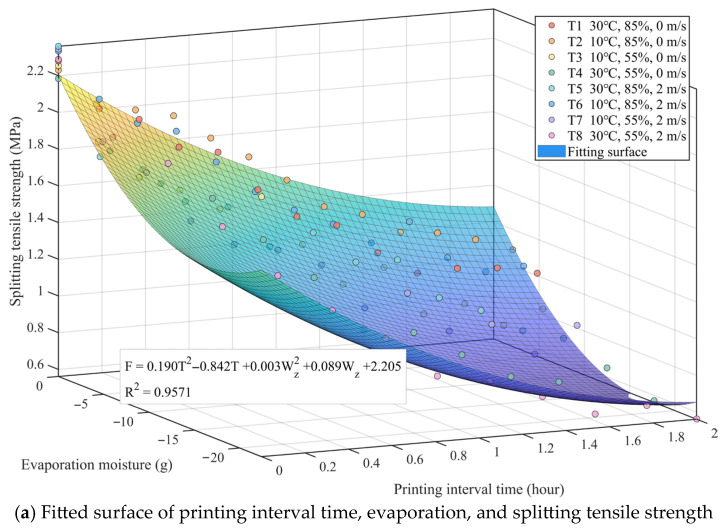

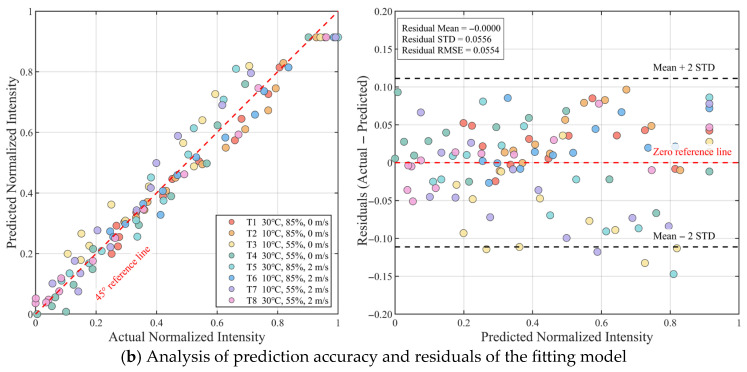
Regression fitting and model validation of concrete splitting tensile strength based on quadratic time term and quadratic evaporation term: (**a**) Fitted surface of printing interval time, evaporation, and splitting tensile strength; (**b**) Analysis of prediction accuracy and residuals of the fitting model.

By substituting the experimental data into the modified quadratic polynomial for regression analysis, the fitted surface and residual distribution of the optimized model were obtained, as presented in Figure 9. The optimized regression equation is given by:

(13) $$\begin{array}{l} F=0.190T^{2}-0.842T+0.003W_{z}^{2}+0.089W_{z}+2.205\\ R^{2}=0.9571 \end{array}$$

Compared to the pre-optimization model, after introducing the quadratic term for evaporation, the coefficient of determination ($R^{2}$) of the model improved from 0.9360 to 0.9571, demonstrating a further enhancement in the model’s capability to capture and characterize the experimental data. From a physical perspective, although the coefficient of the quadratic evaporation term is small (0.003), it is non-negligible. It corrects the non-linear rate of strength decay during stages of severe moisture loss, enabling the model to perform more smoothly and accurately across different environments.

The error statistical results in Figure 9b further confirm the effectiveness of the optimization. Compared with the original model, the root mean square error (RMSE) of the optimized model was reduced from 0.0604 to 0.0554, and the standard deviation (STD) was synchronously reduced to 0.0556. The residual distribution plot reveals that the data points converge more tightly around the zero line, and the degree of dispersion across the entire strength prediction range is significantly improved. This indicates that by introducing the non-linear correction for evaporation, the model not only maintains the original physical mechanism framework but also significantly enhances the robustness and precision of interlayer strength prediction under complex environmental working conditions. This provides more reliable theoretical support for the refined construction control of 3DPC.

### 4.4. Construction of General Model

Although the aforementioned model incorporating the quadratic term significantly improves prediction accuracy, the evaporation (absolute mass) and strength (MPa) parameters in the model still depend on specific specimen dimensions and mix designs, limiting the direct application of the model in different engineering scenarios. To eliminate the influence of differences in specimen geometry and material composition, and to further improve the universality and broad applicability of the model, this paper introduces dimensionless parameters to normalize the model and establish a general model. The Strength Index ($\alpha_{F}$) is defined as:

(14) $$\alpha_{F}=\frac{F(\alpha )}{F_{0,ave}}$$

where F(α) is the predicted strength at any given time, and $F_{0,ave}$ is the average reference strength of the mix proportion under standard curing.

**Figure 10:**
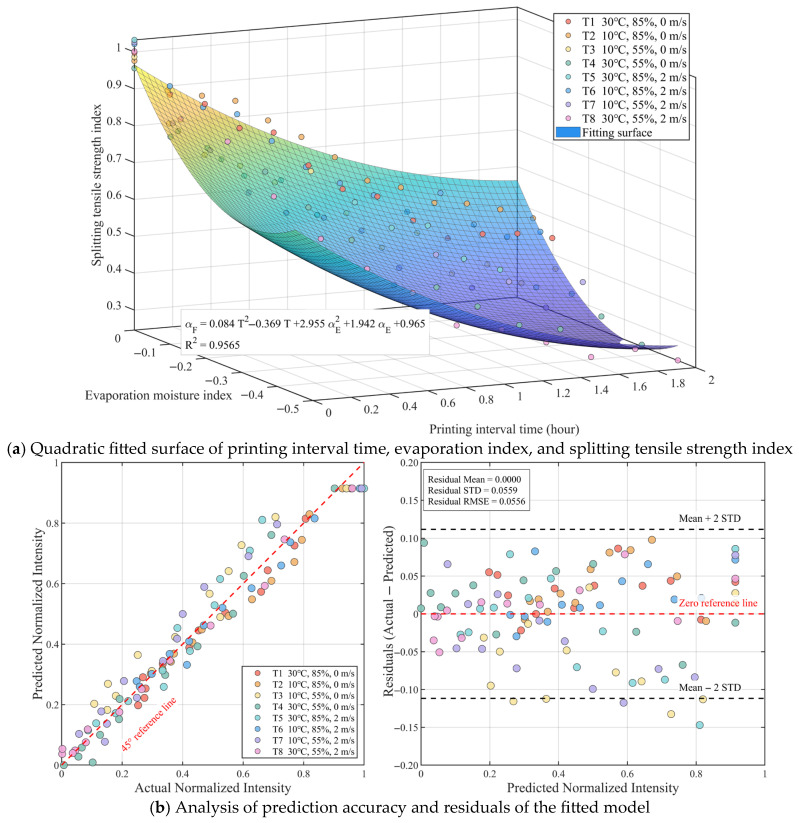
Regression fitting and model validation of concrete splitting tensile strength index based on quadratic time term and quadratic evaporation index term: (**a**) Quadratic fitted surface of printing interval time, evaporation index, and splitting tensile strength index; (**b**) Analysis of prediction accuracy and residuals of the fitted model.

The amount of evaporated water is changed to the evaporation water index $\alpha_{E}$, while the printing interval time T remains unchanged. The general prediction model constructed based on the normalized data is shown in Figure 10. The general equation obtained from non-linear regression is:

(15) $$\begin{array}{l} \alpha_{F}=0.084T^{2}-0.369T+2.955\alpha_{E}^{2}+1.942\alpha_{E}+0.965\\ R^{2}=0.9565 \end{array}$$

As shown in Figure 10a, the coefficient of determination ($R^{2}$) of this general model reaches 0.9565, indicating that after removing the influence of physical dimensions, the model can still excellently capture the intrinsic evolution law among “time–moisture–strength.” From the error analysis in Figure 10b, the root mean square error (RMSE) of the residuals between the predicted strength index and the measured values is only 0.0556, and the residuals exhibit good random distribution characteristics near the zero line.

In the general model, by converting the absolute evaporation amount into the relative evaporation index, the model successfully links the environmental influence factors with the initial water content (deduced from mix proportion) of the material itself. This means that the prediction model is no longer limited to specific printed specimen sizes or specific concrete formulations, but provides a generalized evaluation standard based on the degree of water loss. For 3D-printed components of different sizes or mix proportions, as long as their initial water content and reference strength are known, and after basic calibration of the equation parameters, this equation can be used to make rapid and reliable estimations of the interlayer bond performance under complex evaporation environments.

### 4.5. Engineering Implementation Scheme

In the actual engineering practice of 3DPC, the construction site environment is complex and variable. Factors such as low humidity, high wind speed, and high temperature directly affect the surface moisture evaporation of the printed filaments, leading to fluctuations in interlayer bond strength. Traditional destructive testing methods are lagging and difficult to reflect the real-time quality of the overall structure. Therefore, it is particularly important to construct a non-destructive monitoring system capable of sensing the environment in real-time and predicting structural performance. Based on the established prediction model for interlayer splitting tensile strength of 3DPC, this study proposes an engineering application scheme integrating environmental monitoring, data acquisition and calculation, and digital twin visualization, aiming to solve quality control problems during the printing process.

**Figure 11:**
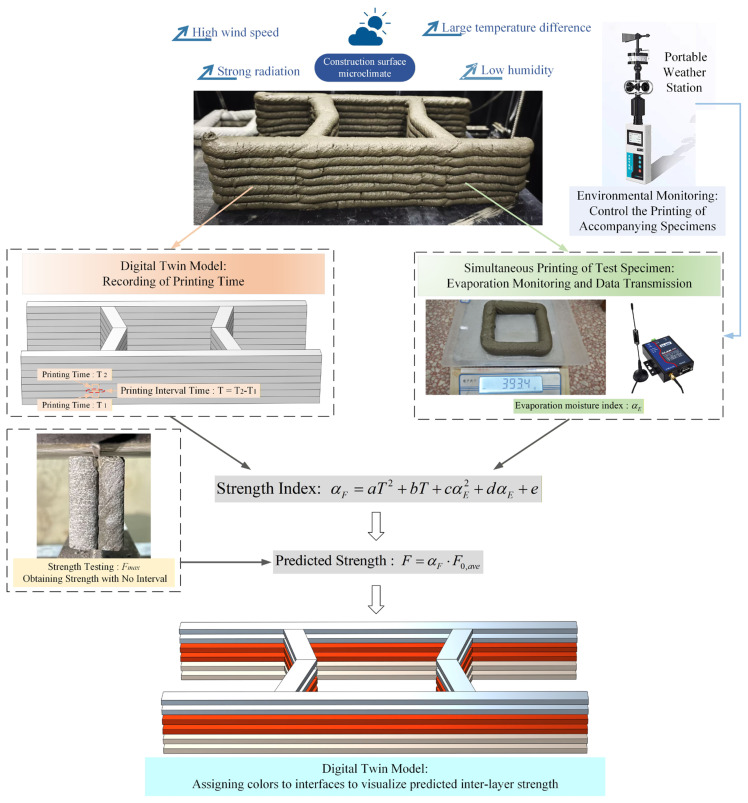
Schematic diagram of the digital twin model for interlayer strength of 3DPC based on monitoring of printing interval time and evaporation.

As shown in Figure 11, the implementation system of this scheme mainly consists of three core parts working in synergy: (1) process data acquisition of environment; (2) printing time, evaporated water amount, and strength prediction calculation; (3) visualization feedback. As illustrated, while printing the main structure, a portable weather station is deployed at the construction site to monitor real-time microclimate data. When significant changes occur in meteorological data, the printing of small companion specimens is triggered. These small companion specimens are printed synchronously alongside the main structure, and the evaporation index ($\alpha_{E}$) is obtained through real-time weighing. This design ingeniously solves the problem of acquiring real evaporation data without interfering with the main printing process. In addition, the digital system automatically records the printing timestamp of each layer, accurately obtaining the printing interval time (T) at any position by calculating the time difference between adjacent layers. Before the printing task begins, only one standard interlayer splitting test is required to determine the reference splitting strength without interval time ($F_{0,ave}$), providing basic data for predicting the actual splitting tensile strength.

In actual use, the system follows data-driven operation logic. The system substitutes the real-time collected evaporation index and printing interval time into the bivariate regression model proposed in this study ($\alpha_{F}=aT^{2}+bT+c\alpha_{E}^{2}+d\alpha_{E}+e$) to calculate the strength index of each interlayer. Then, combining with the reference strength, the predicted tensile strength (F) of the current interface is calculated. Finally, these calculation results are mapped back to the digital twin model in real-time, rendering the printing path through color-coding technology—different colored layers intuitively characterize the distribution differences in predicted strength (e.g., red represents high-strength areas, and blue represents potential weak zones). This visualization method allows engineers to instantly identify structural weak links caused by sudden environmental changes or excessive long intervals, thereby achieving a transformation from post-event inspection to process early warning, and enhancing the safety and reliability of 3DPC engineering.

### 4.6. Limitation

In terms of experimental methodology, despite the rapid operations and standardized procedures adopted during specimen printing and transfer, even brief exposure during the highly sensitive early-age hydration of concrete may cause transient disturbances to the surface moisture state and temperature of the specimens. Specifically, splitting tensile specimens, after the first layer is printed, need to be removed from the environmental chamber, repositioned on the printing platform for the second layer, and then returned to the environmental chamber, thus undergoing an additional disturbance compared to specimens for continuous monitoring. However, considering that the printing and transfer times are relatively short compared to the overall time interval, and all operations are consistent, this study believes that such transient disturbances have a limited and controllable overall impact on the long-term evaporation, dielectric constant evolution, and final interlayer bond strength. Future research could further eliminate such potential disturbances by optimizing experimental equipment, such as directly setting up the 3D printing equipment in a controlled environmental chamber, to obtain more precise experimental data.

Regarding the experimental environmental conditions, although the environmental parameters selected in this study are relevant to actual construction, the experimental conditions necessitated the use of small-scale environmental chambers for staged environmental exposure. This approach could not fully replicate the continuous and highly variable environmental conditions found on actual construction sites. It is important to note that numerous complex environmental factors exist on real construction sites, such as wind speed, solar radiation intensity, and spatial variability of microclimates. These factors can significantly influence the concrete’s moisture evaporation rate, internal hydration kinetics, and ultimately, the interlayer bonding performance. For instance, high wind speeds accelerate moisture loss, and solar radiation causes localized temperature increases, accelerating reaction rates. Both can lead to premature drying of the printed layer interfaces, thereby weakening bond strength. Furthermore, microclimate differences across various areas of a construction site can also lead to non-uniformity in interlayer bonding performance. Therefore, when directly extending the experimental results and prediction models obtained under laboratory conditions in this study to the complex and variable on-site construction environment, their limitations must be carefully considered. Although the model developed in this study exhibits good predictive capabilities on laboratory data, in practical applications, the aforementioned unconsidered environmental factors and constantly changing environmental conditions may affect the model’s prediction accuracy. Future research should consider incorporating more environmental parameters, such as wind speed and solar radiation, into the experimental design. Moreover, developing more comprehensive on-site environmental monitoring techniques, combined with digital twin platforms, could enable real-time, accurate prediction and control of 3D-printed concrete interlayer bonding performance under complex field conditions, thereby further enhancing the model’s generalization ability and practical engineering guidance value.

Regarding the influence of printing parameters, all experiments in this study were based on a single concrete mix proportion, one 3D printer, and fixed layer geometry. Although we proposed a dimensionless model to describe the interlayer bonding performance, its fitted relationships and coefficients have not been fully validated for their generality and extrapolation to other materials, printing systems, or different layer geometries. The sensitivity of the research results may manifest in several aspects: different mix proportion components (e.g., type of cementing material, aggregate gradation, or water-cement ratio) significantly affect the rheological properties, hydration rate, and drying shrinkage of concrete, thereby altering interlayer moisture transport and interfacial bond strength; rheological parameters such as thixotropy and yield stress directly influence the quality of layer deposition and the effectiveness of interlayer contact, thus affecting interlayer bonding; furthermore, changes in nozzle size and printing speed can alter the geometric accuracy, surface roughness, and interlayer contact area of the printed layers. Therefore, the model proposed in this study has certain limitations when applied to materials, printing parameters, or geometries with significant differences. Future work will focus on expanding the experimental scope by introducing various mix proportions, printing equipment, and geometric parameters for validation, and thoroughly investigating the sensitivity of different factors to interlayer bonding performance, with the aim of developing a more universal and robust prediction model.

Regarding the influence of printing paths, this study primarily employed simplified, linear printing paths when evaluating interlayer bond strength. However, actual 3D-printed concrete paths often involve complex geometric features such as curves, directional changes, start-stop events, and overlaps. These complexities can significantly affect extrusion stability, local compaction, surface roughness, and the effective interlayer contact area. For instance, research by Daneshvar et al. [35] showed that curved trajectories are more prone to geometric errors and more sensitive to printing speed compared to straight trajectories. Li et al. [36] also pointed out that printing instability on non-planar surfaces primarily arises from inaccurate fiber deposition, and nozzle height and the decomposition of material self-weight along inclined surfaces are crucial for print quality. These complex paths and the resulting local time delays can cause spatial heterogeneity in interlayer bonding performance under the same environmental conditions, and interact more complexly with the moisture evaporation and hydration kinetics considered in this study. Therefore, the model proposed in this study is primarily based on the decay mechanism of interlayer performance under simplified paths, and its direct applicability to more complex printing trajectories is limited. Future work will consider expanding the scope of research to experimentally investigate the effects of different complex printing paths (e.g., curves, start-stop events, and overlaps) on extrusion quality, interface morphology, and interlayer bonding performance, and strive to develop a more comprehensive model that can incorporate path geometric complexity and the spatial heterogeneity it introduces. This is equally important for improving the reliability of 3D-printed concrete in practical engineering applications.

Regarding model setup, the mechanistic model in this study assumes that moisture evaporation and hydration reactions are decoupled when analyzing interlayer bonding performance during printing intervals. The rationale behind this simplifying assumption is that, under normal environmental conditions and within shorter printing intervals, the internal moisture of the printed body is relatively abundant, and evaporation primarily removes free water with limited overall impact on the cement hydration reaction process. Under this assumption, the model can effectively focus on the critical roles of moisture migration and hydration in interface formation. However, we recognize the potential limitations of this assumption. Particularly under extreme environmental conditions such as high temperature, low humidity, or strong wind, the rate of moisture evaporation significantly accelerates. In such cases, rapid evaporation not only removes surface free water but also accelerates the migration of internal moisture to the surface, leading to insufficient water supply for cement hydration, and even inducing self-desiccation, thereby directly affecting the progress and extent of the hydration reaction. In this scenario, moisture evaporation and hydration reactions are no longer simply decoupled processes but exhibit strong coupling, where evaporation directly limits the progress of hydration reactions. Therefore, this model may have predictive biases under these extreme environments. Since the model does not fully consider the inhibitory effect of rapid moisture loss on the hydration rate, it might overestimate the degree of hydration at printed layer interfaces and the resulting bond strength under high temperature or low humidity conditions. Future research will consider a more comprehensive model of coupled evaporation and hydration, especially under extreme environmental conditions, by incorporating dynamic moisture balance and hydration kinetics equations to improve the model’s universality and predictive accuracy.

## 5. Conclusions

In this paper, full-factorial experiments and theoretical analyses were conducted on the evolution laws of interlayer bonding performance of 3D-printed concrete (3DPC) under complex environments, establishing a physical analysis framework from moisture state to macroscopic mechanical properties. The main conclusions are as follows:

The analysis based on the SHAP interpretable algorithm quantified the degree of influence of the environment and printing interval time on interlayer strength. The results indicate that the extension of printing interval time is the dominating factor leading to interlayer strength decay, with a contribution rate of 68.6%. Among environmental loads, relative humidity is the primary influencing factor (contribution rate 21.3%), far exceeding temperature (6.7%) and wind speed (3.4%). The interlayer splitting tensile strength shows a significant non-linear declining trend with extended interval time, but environmental humidity plays a key regulating role in this decay process. Under extreme conditions of high temperature and low humidity, the interlayer strength falls below the engineering reference threshold of 1.6 MPa within 20–30 min. However, in a high-humidity and windless environment, the strength can remain above 1.6 MPa even with an interval of 40–50 min. This confirms that environmental humidity can effectively compensate for strength loss caused by time delay by inhibiting moisture loss. This suggests that in practical engineering, compared to solely pursuing temperature control, rational planning of the printing rhythm and focusing on regulating environmental humidity are more critical strategies for guaranteeing interlayer quality.

The study further revealed the physical mechanism by which moisture loss leads to the loss of interfacial plasticity and consequently strength decay. Longer printing intervals cause the interface to lose plasticity prematurely due to partial hydration, physically hindering the penetration of hydration products from the subsequent deposited layer. Environmental factors significantly regulate this process mainly by altering the surface evaporation flux; specifically, a high-humidity environment can effectively inhibit moisture loss and delay the interfacial drying and hardening time, thereby exerting a significant compensation effect on strength loss induced by time delay. Monitoring results of dielectric constant and evaporated water amount confirm that the rate of change in the moisture content of the lower layer directly determines the evolution path of interlayer bond strength. Therefore, maintaining an appropriate wetting state at the interface is the physical key to delaying the decay of interlayer bonding force and extending the effective printable time window.

Finally, this study constructed a dimensionless general prediction model with clear physical significance and an engineering application scheme. Based on the correlation mechanism among environment, moisture, and strength, a preliminary regression model containing the quadratic term of printing interval time and linear terms of surface dielectric constant and cumulative evaporation was first constructed, achieving a goodness of fit of 0.93. To further eliminate the specimen size effect and enhance model generalization, the study introduced a normalized evaporation index and established a dimensionless general prediction model containing quadratic terms of both printing interval time and evaporation index. This optimized model improved the goodness of fit to 0.9565, achieving precise quantification of interlayer strength evolution under the action of multiple factors. Accordingly, the paper proposed an engineering application scheme based on the digital twin concept. By monitoring real-time evaporation data of small companion specimens at the construction site and substituting them into the general model combined with printing timestamps, dynamic inversion and visualization warning of the interlayer quality of the main structure can be realized. This method provides an operable theoretical basis and technical path for solving the construction quality control problems of 3DPC under complex climatic environments.

## Figures and Tables

**Table 1:** Chemical composition of cement (wt. %).

Chemical Composition	SiO_2_	Al_2_O_3_	Fe_2_O_3_	CaO	MgO	SiO_3_	Na_2_Oeq
Content	20.54	4.78	3.38	62.58	3.60	1.98	0.59

**Table 2:** Mix proportion of 3DPC (kg/m^3^).

Cement	Sand	Water	Silica Sol	Superplasticizer (PCE)
1430	465	470	28	2.9

**Table 3:** Specifications of main instruments and equipment.

Instruments/Equipment Name	Model	Manufacturer
Cement-based material 3D printing system	Moore 600	Shenzhen Chuangxinyuan Technology Co., Ltd., Shenzhen, China
Programmable constant temperature and humidity chamber	ST-1000LC	Xiamen Espec Instrument Co., Ltd.
Three-parameter portable measuring instrument	PTU310X_10	Jiangsu Tiannuo Base Industry
Microcomputer-controlled electronic universal testing machine	WDW-10D	Jinan Chuance Testing Equipment Co., Ltd.
Ducted fan	12 V 16.5 A	EBM-PAPST, Mulfingen, Germany
Anemometer	RA310	SOKKI, Japan
High-precision digital TDR sensor	TDR-315H	Acclima, Meridian, USA

**Table 4:** Combination scheme of temperature, humidity, and wind speed settings.

Group	Temperature (°C)	Humidity (%)	Wind Speed (m/s)
T1	30	85	0
T2	10	85	0
T3	10	55	0
T4	30	55	0
T5	30	85	2
T6	10	85	2
T7	10	55	2
T8	30	55	2

**Table 5:** Interval printing time setting.

Printing Interval Time Points (min)												
0	10	20	30	40	50	60	70	80	90	100	110	120

**Table 6:** Ranking of characteristics and SHAP importance contribution rates.

Ranking	Feature	Importance Contribution Rate	Percentage
1	Time	0.4254	68.6%
2	Humidity	0.1318	21.3%
3	Temperature	0.0415	6.7%
4	Wind speed	0.0211	3.4%

## Data Availability

The original contributions presented in this study are included in the article. Further inquiries can be directed to the corresponding authors.
